# Prevalence, care-seeking, and health service utilization for non-communicable diseases among Syrian refugees and host communities in Lebanon

**DOI:** 10.1186/s13031-016-0088-3

**Published:** 2016-10-19

**Authors:** Shannon Doocy, Emily Lyles, Baptiste Hanquart, The LHAS Study Team, Michael Woodman

**Affiliations:** 1 Johns Hopkins Bloomberg School of Public Health, 615 N Wolfe St, Suite E8132, Baltimore, MD 21205 USA; 2 Medecins du Monde, Beirut, Lebanon; 3 United Nations High Commissioner for Refugees, Beirut, Lebanon

**Keywords:** Syria, Lebanon, Refugee, Host community, Humanitarian assistance, Non-communicable disease, Chronic disease

## Abstract

**Background:**

Given the large burden of non-communicable diseases (NCDs) among both Syrian refugees and the host communities within which they are settled, humanitarian actors and the government of Lebanon face immense challenges in addressing health needs. This study assessed health status, unmet needs, and utilization of health services among Syrian refugees and host communities in Lebanon.

**Methods:**

A cross-sectional survey of Syrian refugees and host communities in Lebanon was conducted using a two-stage cluster survey design with probability proportional to size sampling. To obtain information on chronic NCDs, respondents were asked a series of questions about hypertension, cardiovascular disease, diabetes, chronic respiratory disease, and arthritis. Differences in household characteristics by care-seeking for these conditions were examined using chi-square, t-test, and adjusted logistic regression methods.

**Results:**

Over half (50.4 %) of refugee and host community households (60.2 %) reported a member with one of the five NCDs. Host community prevalence rates were significantly higher than refugees for all conditions except chronic respiratory diseases (*p* = 0.08). Care-seeking for NCDs among refugees and host community households was high across all conditions with 82.9 and 97.8 %, respectively, having sought care in Lebanon for their condition. Refugees utilized primary health care centers (PHCC) (57.7 %) most often while host communities sought care most in private clinics (62.4 %). Overall, 69.7 % of refugees and 82.7 % of host community members reported an out-of-pocket consultation payment (*p* = 0.041) with an average payment of US$15 among refugees and US$42 for the host community (*p* <0.001).

**Conclusions:**

Given the protracted nature of the Syrian crisis and the burden on the Lebanese health system, implications for both individuals with NCDs and Lebanon’s health system are immense. The burden of out of pocket expenses on persons with NCDs are also substantial, especially given the tenuous economic status of many refugees and the less affluent segments of the Lebanese population. Greater investment in the public sector health system could benefit all parties. Efforts to improve quality of care for NCDs at the primary care level are also a critical component of preventing adverse outcomes and lowering the overall cost of care for NCDs.

## Background

Since the outset of the Syrian conflict in March 2011, an estimated 4.6 million Syrians have fled to neighboring countries [1]. Approximately 1.1 million of these refugees are currently settled in Lebanon, making it the host country with the highest number of refugees per capita in the world [2]. Unlike other countries in the region, Lebanon has not established formal refugee camps for Syrians; instead, refugees are dispersed among villages and communities across the country [3]. With refugees now accounting for one in four people in Lebanon, the burden of the increased population on the country’s highly fragmented and privatized health system is immense [4, 5].

In response to the Syrian crisis, the United Nations High Commissioner for Refugees (UNHCR) established an inter-agency mechanism with the Lebanese Government to coordinate the humanitarian response across all sectors. In the field of health, assistance for Syrian refugees is based on the primary health care strategy, subsidizing primary healthcare services for refugees in existing primary health care centers (PHCC) across the country’s governorates [6–8]. A private sector third party administrator manages referrals for secondary and tertiary services, predominantly covering life-saving emergencies, delivery, and care for newborns [9].

Both Lebanese and Syrian populations underwent the epidemiologic transition from communicable, maternal, neonatal, and nutritional conditions to non-communicable diseases (NCDs) in the past decades [10–12]. As such, both host and refugee populations suffer from a large burden of NCDs, which are often difficult and expensive to manage and require continuity of care to mitigate long-term complications [13–15]. The national and international communities face immense challenges addressing the needs of affected populations in both refugee and host communities due to the high burden of NCDs among refugees and the host country population, the complexity of managing these conditions, and the limited resources available for refugee health care [10, 15–17]. In light of this, we undertook this study to assess the health status, unmet needs, and access to and utilization of health services for NCDs among Syrian refugees and host communities in Lebanon.

## Methods

A survey of Syrian refugees and Lebanese host communities was conducted in March and April 2014. Our primary objectives were to characterize health seeking behaviors and health service access. A cluster design with probability proportional to size sampling was used to attain a nationally representative sample of Syrian refugees living outside of camps. Sample size was determined for key objectives based on the most conservative prevalence estimate of 50 %; calculations assumed 80 % power and a design effect of 2.0. The planned sample was increased from the minimum identified size of 900 refugee households to 1400 refugee households and 700 Lebanese host community households to provide increased precision of point estimates and additional power.

**Fig. 1:**
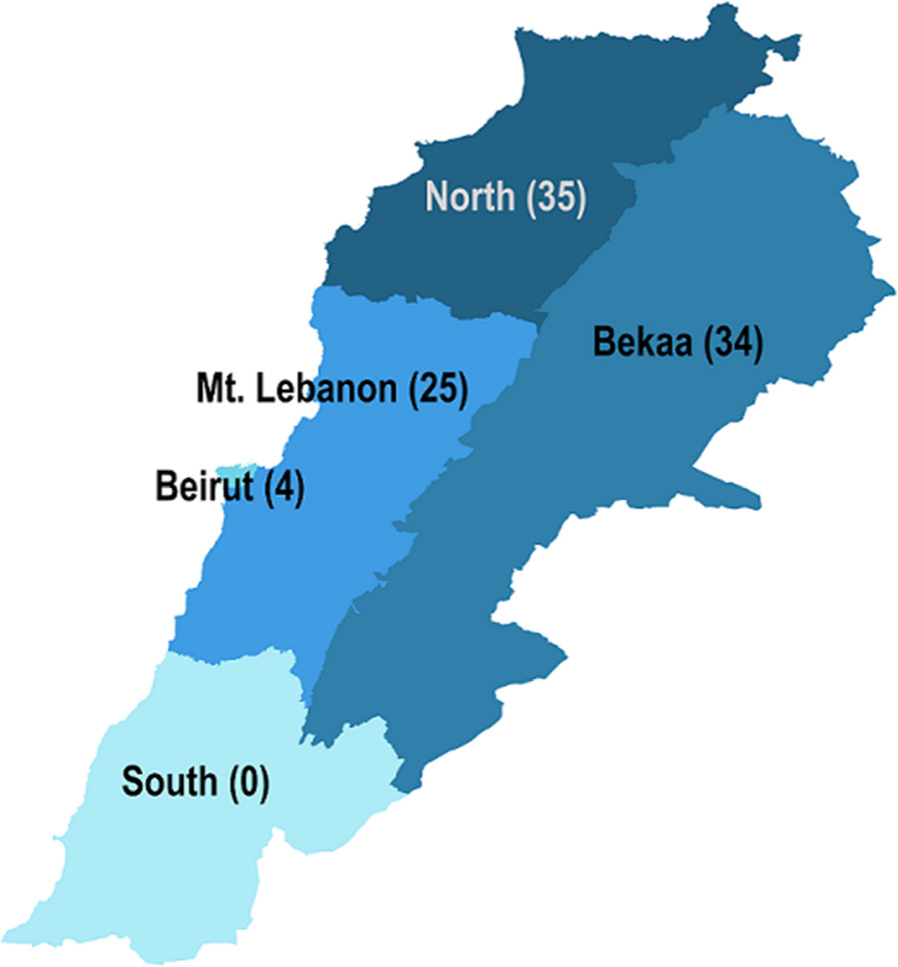
Cluster allocation by Governorate

Given the concentration of Syrian refugees and the low cost of visiting many locations due to the country’s small size, a 100 cluster × 21 household (14 Syrian refugee households and seven host community households) design was used. Probability proportional to size sampling using UNHCR registration data was used to assign clusters to cadastrals, assuming that non-registered refugees had similar residence patterns. Permission to survey in certain security sensitive areas as planned could not be attained which necessitated a re-draw of 28 clusters assigned to 22 inaccessible cadastrals. Clusters were re-assigned to accessible areas using probability proportional to size sampling. The final cluster assignment included 35 clusters (35 %) in the North governorate, 34 clusters (34 %) in Bekaa governorate, 25 clusters (25 %) in Mount Lebanon governorate, four clusters (4 %) in Beirut governorate, and two clusters (2 %) in the South governorate (Fig. 1). Only two cadastrals in the South were accessible to the survey team; presenting data from only these locations was not sufficiently representative of the governorate and would violate cluster sampling assumptions, thus the two South governorate clusters were excluded from the analysis.

ARC GIS software was used to randomly allocate cluster start points within cadastrals. Coordinates in populated areas were used and the nearest intersection to the start point, usually within a half kilometer, was used as the starting survey location. Teams were provided with coordinates, and satellite imagery maps and were instructed to navigate to start points using mapping software such as Google Maps. At the start location, interviewer pairs were sent in different directions to locate households; they approached the nearest business likely to be used by refugees and asked to be referred to nearby Syrian households. Other key informants, notably community residents, were used when there were no nearby shops. Informed verbal consent was sought at the beginning of the interview and only consenting households were interviewed. When interviewers reached a household that consented to participate, the first interview in the cluster was conducted; upon completion, respondents were asked for a referral or introduction to the nearest Syrian household. This referral process was used until 14 Syrian refugee interviews were completed. Following each two completed interviews with Syrian refugee households, interviewers proceeded to the nearest Lebanese household and completed an interview. To improve representativeness and geographic coverage of the sample, no more than three households within the same apartment building were included.

To sample informal tented settlements (ITS), the team estimated the size and area by walking transects and/or the perimeter. When necessary, the ITS was divided into sub-areas of similar size that were assigned to different interviewers. Interviewers located the middle of the settlement/area, spun a pen to randomly select a direction, and then walked in the indicated direction counting the number of shelters passed before reaching the edge of the settlement/area. A randomly selected number between one and the total number of shelters passed was used to identify the starting household. This process was repeated until the necessary number of interviews was complete; referrals were not requested in ITSs to reduce the potential for bias.

Only Syrian households arriving in Lebanon in 2011 or later were eligible to participate, as the aim was to capture the experiences of those displaced by the conflict, so the one approached household that arrived in Lebanon before 2011 was not included in the analysis. Families with both Lebanese and Syrian members were considered Syrian refugees if they arrived in Lebanon in 2011 or later and lived in Syria prior to this time; families who had never lived in Syria were considered as Lebanese host community households for the survey.

The questionnaire was initially developed for use in Jordan and was adapted to the Lebanese context by consensus between partner organizations. Arabic translation of the Jordan questionnaire was adapted for Lebanon and a formal pilot test conducted. The questionnaire focused on health service utilization, access and barriers to care, children’s health, and NCDs. Respondents were asked about the five chronic conditions perceived to be most common among the Syrian refugee population: hypertension, cardiovascular diseases (including heart failure, angina, arrhythmias [irregular heartbeats], a previous heart attack, or previous stroke), diabetes, chronic respiratory diseases (including asthma, chronic bronchitis, emphysema, and chronic obstructive pulmonary disease), and arthritis [18, 19]. Cases were identified through self-reported diagnosis of the condition from a health professional. If more than one household member of any age had a particular condition, one was randomly selected and asked a series of questions on health service utilization and their most recent visit for the chronic health condition.

Interviewers received two days of classroom training that focused on the questionnaire, e-data collection, interview techniques, basic principles of human subjects’ protections and sampling after which two additional days of practical field training were held. To protect the anonymity of respondents, no unique identifiers were recorded and verbal consent was obtained. Interviews lasted between 30 and 60 min depending on the household size, number of children, and individuals with NCDs.

Data was collected on tablets using the Magpi mobile data platform by DataDyne LLC (Washington, DC). Data was analyzed using Stata 13 (College Station, TX) and Tableau Desktop (Seattle, WA) using descriptive statistics and standard methods for comparison of means and proportions. Overall data quality was high. The proportion of missing values was less than 3 % for all variables analyzed, presumably because the questionnaire was designed in the Magpi data collection platform to require responses to these questions. Differences in household characteristics by geographic region, population group (refugee vs. host community), care-seeking, and facility type utilized in Lebanon were examined using chi-square and t-test methods. Characteristics with statistical significance *p* <0.10 in univariate analysis were included in the adjusted logistic regression model to control for confounding. Factors considered potential confounders were chronic condition, region of residence, crowding (more than five household members per sleeping room), highest level of education completed by the household head, socioeconomic quartile (based on monthly expenditures), year of arrival in Lebanon, and receipt of cash, food voucher, non-food voucher, or in-kind assistance. The Stata ‘*svy’* command was used to account for the cluster survey design so that standard errors of the point estimates and model coefficients were adjusted for survey design effects. Amounts in Lebanese Pounds were converted to US dollars at a rate of 1500 LBP per US$1.

The study was approved by the Institutional Review Board at the American University of Beirut. The Johns Hopkins Bloomberg School of Public Health Institutional Review Board also reviewed the protocol and determined that members of the JHSPH team were not involved in human subjects research because they did not have direct contact with participants or access to personal identifiers.

## Results

A total of 2,165 households were approached to participate. Of these, 1.9 % (*n* = 40) were not at home, 0.2 % (*n* = 4) were previously interviewed, 0.05 % (*n* = 1) was ineligible, and 2.7 % (*n* = 58) refused. The final sample included 2,062 households (1,376 Syrian refugee and 686 host Lebanese households), which equates to a response rate of 93.6 %. A total of 1,376 Syrian refugee households participated in the survey with the following age distribution: 7.5 % under 2 years, 13.5 % 2 to less than 5 years, 32.0 % 5–17 years, 33.1 % 18–39 years, 11.1 % 40–59 years and 2.9 % 60+ years. A total of 686 Lebanese host community households participated in the survey with the following age distribution: 3.4 % under 2 years, 6.1 % 2 to less than 5 years, 22.1 % 5–17 years, 35.9 % 18–39 years, 22.2 % 40–59 years and 10.3 % 60+ years.

### Prevalence of non-communicable diseases

**Fig. 2:**
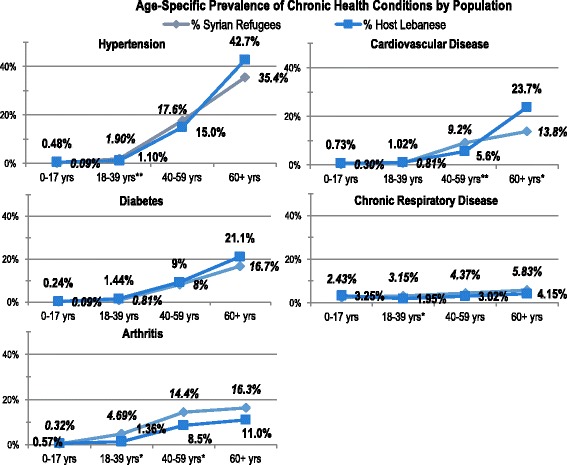
Age-specific prevalence of chronic health conditions by population. * population comparison *p*-value for age group <0.001 ** population comparison *p*-value for age group <0.05

Just over half (50.4, 95 % CI: 47.3–53.6) of surveyed refugee households reported had member(s) previously diagnosed with one of the five included NCDs (hypertension, cardiovascular disease, diabetes, chronic respiratory diseases, and arthritis), significantly lower than host community households (60.2, 95 % CI: 56.5–63.8; population comparison *p* <0.001).^1^ Among adult refugees, arthritis prevalence was the highest (7.9, 95 % CI: 6.8–9.0), followed by hypertension (7.4, 95 % CI: 6.6–8.3), chronic respiratory diseases (3.8, 95 % CI: 3.0–4.5), cardiovascular disease (3.3, 95 % CI: 2. 7–3.9), and diabetes (3.3, 95 % CI: 2.6–3. 9). As compared to refugees, host community prevalence rates were significantly different for all conditions except chronic respiratory diseases (*p* = 0.08). Hypertension was the most prevalent host community condition (10.7, 95 % CI: 9.3–12.0), followed by diabetes (6.3, 95 % CI: 4.2–7.4), arthritis (5.2, 95 CI: 4.3–6.2), cardiovascular disease (5.3, 95 % CI: 4.2–6.4), and chronic respiratory disease (2.6, 95 % CI: 1.9–3.3). Age-specific prevalence of surveyed NCDs is presented in Fig. 2. In younger adults age 18–39 years, arthritis (4.7, 95 % CI: 3.9–5.5) was most prevalent among refugees whereas chronic respiratory disease was most prevalent among host community members (2.0, 95 % CI: 1.1–2.8). Prevalence of all reported NCDs increased substantially after age 40 in both populations (Table 1 & Fig. 2). Hypertension was the most prevalent condition in the 40–59 and 60+ age categories both in the refugee and host community populations. Among children, NCDs were uncommon; chronic respiratory diseases were the most prevalent among children in the refugee (2.4, 95 % CI: 1.8–3.1) and host community populations (3.3, 95 % CI: 1.9–4.6).

**Table 1:** Age-specific chronic disease prevalence

	Hypertension		Cardiovascular disease		Diabetes		Chronic respiratory dis.		Arthritis	
	%	(95 % CI)	%	(95 % CI)	%	(95 % CI)	%	(95 % CI)	%	(95 % CI)
Households where any member(s) have condition										
*Syrian refugees (n = 1376)*	20.5 %	(18.2–23.0)	10.8 %	(9.3–12.6)	9.9 %	(8.2–11.9)	16.0 %	(14.1–18.1)	21.2 %	(18.7–24.0)
*Host community (n = 686)*	34.4 %	(30.8–38.1)	19.8 %	(17.0–23.0)	21.0 %	(18.2–24.1)	12.1 %	(9.8–14.8)	17.2 %	(14.6–20.2)
*p*-value	<0.001		<0.001		<0.001		0.062		0.110	
Prevalence by age group										
*0–17 years*										
*Syrian refugees (n = 4371)*	0.1 %	(0.0–0.2)	0.3 %	(0.1–0.5)	0.1 %	(0.0–0.2)	2.4 %	(1.8–3.1)	0.3 %	(0.1–0.5)
*Host community (n = 1041)*	0.5 %	(−0.2–1.2)	0.7 %	(0.1–1.3)	0.2 %	(−0.2–0.7)	3.3 %	(1.9–4.6)	0.6 %	(−0.1–1.2)
*p*-value	0.261		0.204		0.755		0.526		0.352	
*18–39 years*										
*Syrian refugees (n = 2731)*	1.9 %	(1.4–2.4)	0.8 %	(0.5–1.1)	0.8 %	(0.4–1.2)	3.2 %	(2.5–3.85)	4.7 %	(3.9–5.5)
*Host community (n = 1180)*	1.1 %	(0.5–1.7)	1.0 %	(0.4–1.6)	1.4 %	(0.5–2.4)	2.0 %	(1.1–2.8)	1.4 %	(0.7–2.1)
*p*-value	0.227		0.674		0.123		0.258		<0.001	
*40–59 years*										
*Syrian refugees (n = 915)*	17.6 %	(15.1–20.1)	9.2 %	(7.3–11.1)	8.1 %	(6.1–10.1)	4.4 %	(3.0–5.7)	14.4 %	(11.8–17.0)
*Host community (n = 729)*	15.0 %	(12.5–17.5)	5.6 %	(3.9–7.4)	9.2 %	(7.1–11.3)	3.0 %	(1.7–4.3)	8.5 %	(6.5–10.5)
*p*-value	0.137		0.075		0.253		0.134		0.006	
*60+ years*										
*Syrian refugees (n = 240)*	35.4 %	(29.4–41.4)	13.8 %	(9.9–17.6)	16.7 %	(10.9–22.4)	5.8 %	(2.1–9.6)	16.3 %	(10.4–22.1)
*Host community (n = 337)*	42.7 %	(37.4–48.1)	23.7 %	(19.4–28.1)	21.1 %	(18.1–24.1)	4.2 %	(2.0–6.3)	11.0 %	(7.3–14.6)
*p*-value	0.054		0.001		0.152		0.386		0.140	
*Adult prevalence* ^a^										
*Syrian refugees (n = 3886)*	7.4 %	(6.6–8.3)	3.3 %	(2.7–3.9)	3.3 %	(2.6–3.9)	3.8 %	(3.0–4.5)	7.9 %	(6.8–9.0)
*Host community (n = 2246)*	10.7 %	(9.3–12.0)	5.3 %	(4.2–6.4)	6.3 %	(4.2–7.4)	2.6 %	(1.9–3.3)	5.2 %	(4.3–6.2)
*p*-value	<0.001		<0.001		<0.001		0.080		0.028	

^a^ “Adult” defined as individual over 17 years old

### Care-seeking for non-communicable diseases

Care-seeking for NCDs among Syrian refugees and Lebanese host community households was high across all conditions. Of the 1,079 Syrian refugee cases with an NCD diagnosis, 82.9 (95 % CI: 80.2–85.2) received care in Lebanon and 16.2 (95 % CI: 13.9–18.9) reported that care in Lebanon was not needed. With respect to specific NCDs in the refugee population, Syrians with chronic respiratory disease sought care at the highest rate in Lebanon, with 89.1 % (95 % CI: 84.1–92.6) reporting seeing a doctor for the condition in Lebanon. Frequency of refugee care-seeking was highest among diabetics (70.0, 95 % CI: 61.2–77.5 of cases sought care in the past 3 months) and the lowest for cardiovascular disease (49.6, 95 % CI: 39.4–59.8 of cases sought care in the 3 months preceding the survey).

In general, Lebanese host community members were significantly more likely to have sought care in Lebanon for NCDs than refugees; presumably this is a function of length of time in the country. There were no significant differences in the time period since care was last received in Lebanon between refugees and the host community (*p* = 0.118) for NCDs overall (Table 2). Syrians with arthritis sought care had the lowest percentage of care-seeking care at 78.1 % (95 % CI: 73.0–82.4). In contrast to refugees, the lowest proportion of care-seeking in the host community was observed among those with chronic respiratory disease (92.8, 95 % CI: 84.7–96.7) and the highest proportion among those with arthritis, all of which reported care-seeking. Frequency of host community care-seeking was highest among individuals with arthritis (56.8, 95 % CI: 47.6–65.6 of cases sought care in the past 3 months), while the lowest care-seeking rate among host community was for diabetes (32.1, 95 % CI: 25.0–40.2 of cases sought care in the 3 months preceding the survey). Similar proportions of refugees and host community members sought care for their condition within the past 3 months.

**Table 2:** Care seeking for chronic medical conditions among adult refugees and the host community

	All conditions		Hypertension		Cardiovascular disease		Diabetes		Chronic respiratory disease		Arthritis	
	%	(95 % CI)	%	(95 % CI)	%	(95 % CI)	%	(95 % CI)	%	(95 % CI)	%	(95 % CI)
Ever sought care in Lebanon												
*Syrian refugees*	*n* = 1079		*n* = 282		*n* = 149		*n* = 136		*n* = 220		*n* = 292	
	82.9	(80.3–85.3)	80.9	(75.2–85.5)	82.6	(74.8–88.3)	88.2	(81.8–92.6)	89.1	(84.1–92.6)	78.1	(73.0–82.4)
*Host community*	*n* = 717		*n* = 236		*n* = 136		*n* = 144		*n* = 83		*n* = 118	
	97.8	(96.3–98.7)	98.3	(95.6–99.4)	98.5	(94.3–99.6)	97.2	(92.9–98.9)	92.8	(84.7–96.7)	100.0	
*p*-value	<0.001		<0.001		<0.001		0.004		0.256		<0.001	
Most recent careseeking for condition												
*Syrian refugees*	*n* = 895		*n* = 228		*n* = 123		*n* = 120		*n* = 196		*n* = 228	
<1 month ago	29.7	(26.0–33.8)	32.5	(26.6–38.9)	18.7	(11.7–28.6)	37.5	(28.7–47.3)	36.2	(30.0–43.0)	23.2	(18.3–29.1)
1 month to <3 months ago	31.3	(27.9–34.9)	31.1	(25.2–37.8)	30.9	(22.4–40.9)	32.5	(24.5–41.7)	31.1	(24.7–38.4)	31.1	(25.4–37.6)
3 months to <6 months ago	15.8	(13.1–18.8)	16.2	(11.7–22.1)	22.8	(15.7–31.8)	10.8	(6.6–17.2)	14.8	(10.3–20.8)	14.9	(10.6–20.6)
6 months to <1 year ago	6.3	(4.9–8.0)	5.7	(3.2–9.8)	7.3	(4.0–13.2)	4.2	(1.8–9.6)	4.1	(2.1–7.8)	9.2	(6.1–13.7)
>1 year ago	17.0	(14.1–20.3)	14.5	(10.1–20.3)	20.3	(14.0–28.6)	15.0	(9.9–22.1)	13.8	(9.5–19.5)	21.5	(16.5–27.5)
*Host community*	*n* = 701		*n* = 232		*n* = 134		*n* = 140		*n* = 77		*n* = 118	
<1 month ago	23.7	(20.2–27.5)	25.4	(20.5–31.1)	23.9	(17.9–31.1)	26.4	(19.7–34.5)	27.3	(17.6–39.8)	14.4	(8.8–22.8)
1 month to <3 months ago	33.0	(29.2–36.9)	30.6	(25.3–36.5)	31.3	(23.6–40.4)	41.4	(33.3–50.0)	33.8	(24.4–44.6)	28.8	(22.0–36.8)
3 months to <6 months ago	18.0	(15.2–21.1)	18.1	(13.7–23.6)	19.4	(14.0–26.3)	17.1	(11.6–24.6)	18.2	(11.0–28.5)	16.9	(10.4–26.4)
6 months to <1 year ago	8.6	(6.6–11.0)	10.3	(7.1–14.9)	9.7	(5.5–16.6)	5.0	(2.5–9.9)	10.4	(5.1–20.1)	6.8	(3.4–13.0)
>1 year ago	16.8	(13.8–20.4)	15.5	(11.1–21.2)	15.7	(11.1–21.6)	10.0	(5.9–16.4)	10.4	(5.3–19.4)	33.1	(24.8–42.5)
*p*-value	0.118		0.099		0.286		0.800		0.444		0.018	
Most recent careseeking for condition was within the 3 months preceding survey												
*Syrian refugees*	61.0	(56.9–64.9)	63.6	(56.4–70.2)	49.6	(39.4–59.8)	70.0	(61.2–77.5)	67.3	(60.2–73.8)	54.4	(47.9––60.7)
*Host community*	43.4	(39.2–47.6)	44.0	(37.5–50.7)	44.8	(36.4–53.5)	32.1	(25.0–40.2)	39.0	(28.3–50.8)	56.8	(47.6–65.6)
*p*-value	0.095		0.074		0.396		0.687		0.387		0.056	
Facility type utilized for most recent careseeking for condition												
*Syrian refugees*	*n* = 743		*n* = 195		*n* = 98		*n* = 102		*n* = 169		*n* = 179	
PHCC	57.7	(53.2,62.1)	54.9	(47.6,61.9)	44.9	(36.0,54.1)	60.8	(50.7,70.1)	63.3	(55.3,70.6)	60.9	(53.9,67.5)
Private clinic	19.8	(16.9,23.0)	21.5	(16.6,27.5)	17.3	(11.5,25.2)	20.6	(13.7,29.7)	17.2	(12.1,23.8)	21.2	(15.9,27.8)
Pharmacy	9	(6.6,12.2)	9.2	(5.6,14.8)	7.1	(3.6,13.8)	9.8	(5.5,16.8)	8.3	(4.9,13.7)	10.1	(5.9,16.7)
Hospital	8.7	(6.6,11.4)	8.2	(5.1,12.9)	24.5	(17.4,33.3)	3.9	(1.5,9.9)	8.3	(4.9,13.7)	3.9	(1.9,7.9)
Other	4.7	(3.0,7.2)	6.2	(3.0,12.3)	6.1	(2.8,12.7)	4.9	(2.1,11.1)	3	(1.0,8.1)	3.9	(1.9,7.9)
*Host community*	*n* = 583		*n* = 196		*n* = 113		*n* = 126		*n* = 69		*n* = 79	
PHCC	16.6	(13.2,20.8)	19.4	(14.3,25.7)	7.1	(3.6,13.5)	17.5	(12.1,24.5)	14.5	(7.5,26.0)	24.1	(16.2,34.2)
Private clinic	61.6	(55.6,67.3)	59.2	(51.0,66.9)	65.5	(54.8,74.8)	59.5	(51.2,67.3)	69.6	(57.9,79.1)	58.2	(47.6,68.2)
Pharmacy	2.7	(1.5,5.1)	2.6	(1.0,6.1)	1.8	(0.4,6.9)	6.3	(3.3,12.0)	0		1.3	(0.2,8.8)
Hospital	16.6	(12.9,21.2)	15.8	(11.4,21.6)	22.1	(14.7,31.9)	15.9	(10.7,22.8)	13	(7.1,22.8)	15.2	(8.9,24.7)
Other	2.4	(1.2,4.9)	3.1	(1.2,7.5)	3.5	(1.3,9.1)	0.8	(0.1,5.6)	2.9	(0.7,11.3)	1.3	(0.2,8.8)
*p*-value	<0.001		0.006		0.222		<0.001		0.001		0.002	

### Predictors of care-seeking

Results of univariate and multivariate logistic regression analyses for predictors of care-seeking for NCDs among Syrian refugees in Lebanon are presented in Table 3. Refugee odds of seeking care were significantly associated with type of NCD and year of arrival in Lebanon in both univariate and multivariate regression analyses. Refugees with chronic respiratory disease had 1.9 (95 % CI: 1.09–3.31) times higher odds of care-seeking than those with hypertension; no significant differences in care-seeking rates were observed for other conditions. There was an inverse relationship between care-seeking and year of arrival in Lebanon, as would be expected, where newer arrivals (2013 and 2014) had 33 % (95 % CI: 2–55) lower odds of care-seeking than those arriving in 2011 and 2012. Among host community households, only NCD type was significantly associated with care-seeking in both univariate and multivariate regression. Host community members with chronic respiratory disease had 79 % (95 % CI: 30–94) lower odds of care-seeking than those with hypertension; no significant differences in care-seeking rates were observed for other conditions.

**Table 3:** Characteristics by care seeking decision and odds of care seeking among Syrian refugees and host communities in Lebanon^a^

	Syrian refugees						Lebanese host community					
	Overall	Sought care	Did not seek care	*p-value*	Odds of care seeking		Overall	Sought care	Did not seek care	*p-value*	Odds of care seeking	
					Crude OR	Adjusted OR					Crude OR	Adjusted OR
	*(n = 1079)*	*(n = 895)*	*(n = 184)*		(95 % CI)^b^	(95 % CI)^c^	*(n = 717)*	*(n = 701)*	*(n = 16)*		(95 % CI)^b^	(95 % CI)^c^
Chronic condition												
Hypertension	26.1 %	25.5 %	29.3 %	***0.009***	Reference	Reference	32.9 %	33.1 %	25.0 %	***0.001***	Reference	Reference
Cardiovascular disease	13.8 %	13.7 %	14.1 %		1.12 (0.67–1.88)	1.13 (0.67–1.92)	19.0 %	19.1 %	12.5 %		1.16 (0.20–6.55)	1.17 (0.21–6.56)
Diabetes	12.6 %	13.4 %	8.7 %		1.78 (1.00–3.16)	1.65 (0.93–2.93)	20.1 %	20.0 %	25.0 %		0.60 (0.14–2.55)	0.59 (0.14–2.55)
Chronic Pulmonary disease	20.4 %	21.9 %	13.0 %		**1.93 (1.11–3.63)**	**1.90 (1.09–3.31)**	11.6 %	11.0 %	37.5 %		***0.22 (0.07–0.73)***	**0.21 (0.06–0.70)**
Arthritis	27.1 %	25.5 %	34.8 %		0.84 (0.56–1.26)	0.84 (0.55–1.26)	16.5 %	16.8 %	0 %		***---***	***---***
Region of residence												
Beirut/Mt. Lebanon	25.7 %	25.4 %	27.2 %	0.617	Reference		31.7 %	31.1 %	56.2 %	***0.095***	Reference	Reference
Bekaa	34.4 %	34.0 %	36.4 %		1.00 (0.62–1.61)		33.1 %	33.2 %	25.0 %		2.40 (0.73–7.88)	2.54 (0.79–8.19)
North	39.9 %	40.7 %	36.4 %		1.20 (0.76–1.89)		35.3 %	35.7 %	18.8 %		***3.44 (0.89–13.32)***	3.5 (0.89–13.92)
Household characteristics												
Crowding (5+ / sleeping room)	44.9 %	45.0 %	44.5 %	0.920	1.02 (0.71–1.47)		8.7 %	8.9 %	0 %	0.277	***---***	***---***
Household head education (Highest level completed)												
None	18.4 %	18.7 %	16.8 %	***0.018***	Reference	Reference	19.2 %	19.4 %	12.5 %	0.486	Reference	***---***
Primary	38.1 %	39.9 %	29.3 %		1.23 (0.72–2.10)	1.19 (0.69–2.03)	30.0 %	30.2 %	18.8 %		1.04 (0.16–6.76)	***---***
Preparatory	29.4 %	29.1 %	31.0 %		0.85 (0.47–1.53)	0.82 (0.45–1.49)	23.3 %	23.0 %	37.5 %		0.39 (0.08–1.98)	***---***
Secondary or higher	14.2 %	12.4 %	22.8 %		**0.49 (0.24–1.00)**	0.51 (0.25–1.04)	27.5 %	27.4 %	31.2 %		0.56 (0.09–3.54)	***---***
Socioeconomic quartile (based on monthly expenditures)												
Bottom	19.9 %	19.1 %	23.9 %	0.545	Reference		21.5 %	21.4 %	25.0 %	0.743	Reference	***---***
2nd	23.5 %	23.2 %	25.0 %		1.16 (0.70–1.93)		22.5 %	22.7 %	12.5 %		2.12 (0.39–11.61)	***---***
3rd	23.0 %	23.5 %	20.7 %		1.42 (0.76–2.67)		26.4 %	26.4 %	25.0 %		1.23 (0.26–5.76)	***---***
Top	33.5 %	34.2 %	30.4 %		1.41 (0.81–2.45)		29.7 %	29.5 %	37.5 %		0.92 (0.32–2.63)	***---***
Year of arrival in Lebanon												
2011–2012	45.5 %	47.3 %	36.4 %	***0.023***	Reference	Reference						
2013–2014	54.5 %	52.7 %	63.6 %		**0.64 (0.43–0.94)**	**0.67 (0.45–0.98)**						
Assistance received^d^												
Cash	4.1 %	4.0 %	4.3 %	0.898	1.08 (0.31–3.79)							
Food voucher	73.2 %	73.3 %	72.8 %	0.136	0.86 (0.60–1.25)							
Non-food voucher	20.0 %	19.8 %	21.2 %	0.700	1.09 (0.70–1.71)							
Non-food items	11.7 %	11.4 %	13.0 %	0.546	1.17 (0.70–1.93)							

^a^ “care-seeking” defined as having sought care last time it was needed

^b^ bold indicates statistically significant (*p* <0.10) findings

^c^ model only includes variables with statistical significance in the univariate analysis; bold indicates statistically significant (*p* <0.05) findings

^d^ analyzed as dichotomous variables (assistance type received/not received)

### Health facility utilization

**Fig. 3:**
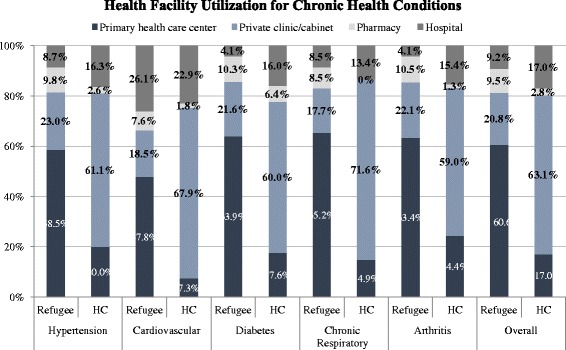
Health facility utilization for chronic health conditions

Significant differences in care-seeking location were observed between refugees and the host community (*p* <0.001) (Fig. 3). Over half of the Syrian refugee care seekers received care in primary health care centers (57.7, 95 % CI: 53.2–62.1) compared to only 16.6 % (95 % CI: 13.3–20.8) of host community care seekers. In contrast, host community cases were most likely to seek care at private clinics (61.6, 95 % CI: 55.6–67.3), which were utilized by a smaller proportion of refugees (19.8, 95 % CI: 16.9–23.0). A smaller proportions of patients also sought care at hospitals (8.7, 95 % CI: 6.6–11.4 of refugee and 16.6, 95 % CI: 12.9–21.2 of host community cases) and pharmacies (9.0, 95 % CI: 6.6–12.2 of refugees and 2.7, 95 % CI: 1.5–5.1 of host community cases); the remaining patients sought care from a number of other sources including mobile medical units (MMUs) and home-based providers (4.7, 95 % CI: 3.0–7.2 of refugee and 2.4, 95 % CI: 1.2–4.9 of host community cases) (Table 2).

Primary health care centers were utilized by the highest proportion of Syrian refugees with chronic respiratory disease (63.3, 95 % CI: 55.3–70.6) as compared to the other four NCDs included in the survey. Cardiovascular cases reported the lowest proportion of refugees utilizing primary health care centers for care (44.9, 95 % CI: 36.0–54.1). Private clinics were utilized by the highest proportion of refugee NCD cases for hypertension care (21.5, 95 % CI: 16.6–27.5) while the lowest proportion of Syria refugees utilizing private clinics did so for chronic respiratory disease care (17.2, 95 % CI: 12.1–23.8). Hospitals were most often utilized by refugees seeking cardiovascular care (24.5, 95 % CI: 17.4–33.3) while the lowest proportion of hospital careseekers did so for diabetes (3.9, 95 % CI: 1.5–9.9) and arthritis care (3.9, 95 % CI: 1.9–7.9). Conversely, pharmacies were utilized the most among Syrian refugee patients with arthritis (10.1, 95 % CI: 5.9–16.7) and least among cases of cardiovascular disease (7.1, 95 % CI: 3.6–13.8).

Among host community households, cardiovascular patients reported the lowest proportion utilizing primary health care centers for care (7.1, 95 % CI: 3.6–13.5) and arthritis patients represented the highest proportion seeking care in PHCCs (24.1, 95 % CI: 16.2–34.2). Private clinics were utilized by the highest proportion of host community individuals with chronic respiratory disease (69.6, 95 % CI: 57.9–79.1) and the lowest proportion of patients with arthritis (58.2, 95 % CI: 47.6–68.2). Hospitals were utilized the most among Lebanese host community patients with cardiovascular disease (22.1, 95 % CI: 14.7–31.9) and least among cases of chronic respiratory disease (13.0, 95 % CI: 7.1–22.8). Among all NCDs included in the survey, the lowest proportion of host community cases utilizing pharmacies was observed for chronic respiratory disease, for which no care was sought in pharmacies, whereas host community pharmacy care seeking was highest for diabetes care (6.3, 95 % CI: 3.3–12.0). No significant differences were observed in care seeker characteristics (UNHCR registration status, household head education attainment, socioeconomic quartile, crowding, year of arrival in Lebanon, region of residence, and specific NCD) by sector where care was sought for either refugees or host community members.

### Spending on health services for non-communicable diseases

**Fig. 4:**
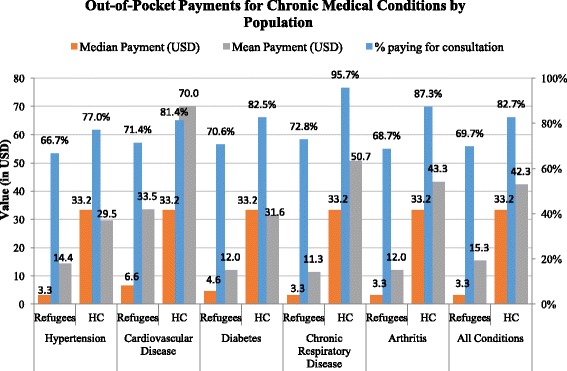
Out-of-pocket payments for chronic medical conditions by population

Cost of care-seeking for NCDs was measured for the most recent care visit. Out-of-pocket payments for the consultation, including diagnostic and laboratory tests, were measured; payments made on the patient’s behalf by the United Nations, insurance, or another organization were excluded as were payments for medication. Out-of-pocket expenditures are presented in Tables 4 and 5 and Fig. 4. Overall, 69.7 % (95 % CI: 65.0–74.0) of refugees and 82.7 % (95 % CI: 77.9–86.6) of host community members reported an out-of-pocket consultation payment (population comparison *p* = 0.041). The average out-of-pocket consultation payment was US$15 (95 % CI: 12.8–17.8; median US$3) for refugees and US$42 (95 % CI: 35.6–49.0; median US$33) for host community members. Among only those that paid for care, the average out-of-pocket payment was US$22 (95 % CI: 18.6–25.5; median US$10) for refugees and US$51 (95 % CI: 43.7–58.7; median US $33) among the host community (population comparison *p* <0.001). No significant differences in the proportion of patients with out-of-pocket payments were observed by region for refugees or host community members (refugee regional comparison *p* = 0.061 and host community regional comparison *p* = 0.984) and average payment amount by region was similar for the host community (host community regional comparison *p* = 0.905). However, the mean out-of-pocket payment amount differed by region for refugees and was significantly higher in Beirut/Mount Lebanon (US$20, 95 % CI: 12.8–28.6) compared to the North, where the lowest average payment (US$12, 95 % CI: 9.8–14.8) was observed (refugee regional comparison *p* = 0.029).

**Table 4:** Out-of-pocket expenditures for chronic disease care in Lebanon (US dollars)^a^

		All NCDs		Hypertension		Cardiovascular disease		Diabetes		Chronic resp. disease		Arthritis		
		*(Refugees n = 895 Host Com. n = 701)*		*(Refugees n = 228 Host Com. n = 232)*		*(Refugees n = 123 Host Com. n = 134)*		*(Refugees n = 120 Host Com. n = 140)*		*(Refugees n = 196 Host Com. n = 77)*		*(Refugees n = 228 Host Com. n = 118)*		
		Point	(95 % CI)	Point	(95 % CI)	Point	(95 % CI)	Point	(95 % CI)	Point	(95 % CI)	Point	(95 % CI)	*p-*value
Paid provider for consultation^b^														
*Syrian refugees*		69.7	(65.0–74.0)	66.7	(59.2–73.4)	71.4	(61.9–79.4)	70.6	(62.3–77.7)	72.8	(64.7–79.6)	68.7	(60.8–75.6)	0.487
*Host community*		82.7	(77.9–86.6)	77.0	(70.7–82.3)	81.4	(72.6–87.8)	82.5	(74.5–88.4)	95.7	(87.5–98.6)	87.3	(77.8–93.1)	0.090
*p*-value (proportion paying)		***0.041***		0.545		0.608		***0.022***		***<0.001***		0.262		
Cost of consultation (USD)^b^														
*Syrian refugees*	Median	3.3		3.3		6.6		4.6		3.3		3.3		
	Mean	15.3	(12.8–17.8)	14.4	(9.8–18.9)	33.5	(19.6–47.4)	12.0	(8.2–15.7)	11.3	(8.3–14.3)	12	(9.5–14.5)	***0.009***
*Host community*	Median	33.2		33.2		33.2		33.2		33.2		33.2		
	Mean	42.3	(35.6–49.0)	29.5	(24.8–34.2)	70.0	(44.0–96.1)	31.6	(26.7–36.5)	50.7	(37.7–63.8)	43.3	(27.6–58.9)	0.198
*p*-value (payment amount)		***<0.001***		***<0.001***		***0.022***		***<0.001***		***<0.001***		***<0.001***		
*Among cases that paid*														
*Syrian refugees*	Median	10		10		13.3		10		6.6		8.3		
	Mean	22.0	(18.6–25.5)	21.7	(14.9–28.5)	47.3	(29.2–65.5)	17.1	(12.0–22.2)	15.6	(11.9–19.2)	17.5	(14.4–20.7)	***0.004***
*Host community*	Median	33.2		33.2		33.2		33.2		33.2		33.2		
	Mean	51.2	(43.7–58.7)	38.4	(33.3–43.5)	85.6	(54.9–116.3)	38.7	(33.3–44.0)	53.1	(39.7–66.6)	49.1	(31.9–66.2)	0.827
*p*-value (payment amount)		***<0.001***		***<0.001***		***0.046***		***<0.001***		***<0.001***		***<0.001***		

^a^ Bold italic indicates statistically significant (*p* < 0.50) findings

^b^ Among those seeking care in Lebanon for condition

**Table 5:** Consultation payments for chronic disease care in Lebanon by facility type utilized (US dollars)^a^

		All NCDs		Hypertension		Cardiovascular disease		Diabetes		Chronic resp. disease		Arthritis		
		*(Refugees n = 895 Host Com. n = 701)*		*(Refugees n = 228 Host Com. n = 232)*		*(Refugees n = 123 Host Com. n = 134)*		*(Refugees n = 120 Host Com. n = 140)*		*(Refugees n = 196 Host Com. n = 77)*		*(Refugees n = 228 Host Com. n = 118)*		
		Point	(95 % CI)	Point	(95 % CI)	Point	(95 % CI)	Point	(95 % CI)	Point	(95 % CI)	Point	(95 % CI)	*p*-value
*Primary health care center*														
Syrian refugees	Median	3.3		3.3		3.6		4.3		3.3		3.3		
	Mean	8.1	(6.4–9.8)	7	(3.3–11.4)	9.1	(4.5–13.6)	10.6	(5.3–16.0)	6.8	(4.2–9.4)	8.3	(5.5–11.0)	0.976
% paying for provider visit^b^		79.0	(72.5–84.3)	74	(63.7–82.0)	84.1	(69.9–92.3)	79	(66.4–87.8)	82.2	(73.0–88.8)	78.9	(68.7–86.4)	0.341
Host community	Median	10.0		10.0		10.0		8.0		14.9		13.3		
	Mean	17.1	(12.3–21.9)	18.8	(10.1–27.5)	9.5	(4.5–14.6)	12.7	(6.2–19.2)	31.0	(8.7–53.2)	14.4	(8.4–20.4)	0.930
% paying for provider visit^b^		82.5	(69.2–90.8)	86.8	(71.7–94.5)	75.0	(37.0–93.9)	77.3	(55.2–90.4)	90.0	(57.8–98.3)	78.9	(55.0–92.0)	0.309
*p*-value (payment amount)		***<0.001***		***0.012***		0.889		0.622		0.037		0.067		
*p*-value (proportion paying)		0.777		0.078		0.581		0.868		0.467		0.391		
*Private clinic (Cabinet)*														
Syrian refugees	Median	26.5		23.2		29.9		19.9		26.5		29.5		
	Mean	27.6	(23.7–31.5)	28.5	(17.5–39.4)	32.8	(16.4–49.1)	24.2	(16.3–32.1)	25.6	(19.5–31.6)	27.9	(22.0–33.7)	0.742
% paying for provider visit^b^		86.4	(79.7–91.1)	85.7	(71.8–93.4)	76.5	(51.0–91.0)	90.5	(69.5–97.5)	86.2	(67.5–95.0)	89.5	(75.0–96.0)	0.462
Host community	Median	33.2		33.2		33.2		33.2		33.2		33.2		
	Mean	39.6	(35.0–44.1)	32.3	(28.0–36.7)	40.2	(32.6–47.7)	41.8	(35.7–47.8)	43.2	(33.6–52.8)	49.3	(31.2–67.3)	0.056
% paying for provider visit^b^		89.7	(85.3–92.9)	82.8	(74.6–88.7)	85.1	(75.0–91.6)	94.7	(86.6–98.0)	97.9	(86.6–99.7)	97.8	(86.1–99.7)	***0.003***
*p*-value (payment amount)		***<0.001***		0.506		0.436		***0.001***		***0.005***		**0.029**		
*p*-value (proportion paying)		0.991		0.144		0.425		0.538		0.097		0.127		
*Hospital*														
Syrian refugees	Median	19.9		19.9		33.2		0		28.2		0		
	Mean	57.8	(37.0–78.5)	48.8	(11.0–86.6)	97.5	(52.1–142.9)	2.2	(−1.4–5.8)	29.9	(15.3–44.5)	25.9	(−1.3–53.2)	***0.024***
% paying for provider visit^b^		63.1	(50.3–74.2)	62.5	(38.9–81.4)	70.8	(50.3–85.3)	50.0	(12.0–88.0)	64.3	(36.2–85.1)	42.9	(14.0–77.5)	0.387
Host community	Median	33.2		33.2		66.3		24.9		99.5		19.9		
	Mean	92.5	(58.2–126.7)	42.3	(22.0–62.7)	201.0	(91.7–310.4)	30.0	(15.8–44.3)	118.5	(54.9–182.1)	73.8	(0.8–146.8)	0.923
% paying for provider visit^b^		77.3	(66.3–85.5)	64.5	(45.6–79.8)	84.0	(66.6–93.3)	80.0	(56.4–92.5)	100		75.0	(44.2–91.9)	0.107
*p*-value (payment amount)		0.105		0.764		0.116		***<0.001***		***0.015***		0.250		
*p*-value (proportion paying)		0.436		0.897		0.716		0.267		***0.016***		0.181		

^a^ Bold italic indicates statistically significant (*p* < 0.50) findings

^b^ As a percent of those seeking care in Lebanon in this facility type for condition

Refugee spending on consultation fees was similar among the five NCDs when measured by the proportion of patients with an out-of-pocket consultation payment (refugee cross-condition comparison *p* = 0.487), but varied significantly in the amount of payment (cross-condition comparison *p* = 0.009). The highest average payment among refugees was observed for cardiovascular disease (mean US$34, 95 % CI: 19.6–47.4; median US$7) and the lowest for chronic respiratory disease (mean US$11, 95 % CI: 8.3–14.3; median US$3). While no statistically significant differences in proportion of refugee respondents with payments by facility type were observed (private clinics, 86.4, 95 % CI: 79.7–91.1; PHCCs, 79.0, 95 % CI: 72.5–84.3; and hospitals, 63.1, 95 % CI: 50.3–74.2; *p* = 0.100), the mean payment amount by refugees differed significantly by facility type and was higher in hospitals (US$58, 95 % CI: 37.0–78.5) compared to private clinics (US$28, 95 % CI: 23.7–31.5) and PHCCs (US$8, 95 % CI: 6.4–9.8) (facility type comparison among refugees *p* <0.001).

Unlike refugees, mean out-of-pocket payment amounts were similar for all conditions in host community cases (cross-condition comparison *p* = 0.198), as was the proportion visits with an out-of-pocket consultation payment (cross-condition comparison *p* = 0.090). Among the host community, the proportions with out-of-pocket payments were also similar across facility types (private clinics, 89.7, 95 % CI 85.3–92.9; PHCCs, 82.5, 95 % CI: 69.2–90.8; and hospitals, 77.3, 95 % CI: 66.3–85.5; facility type comparison *p* = 0.311); however, differences in the average payment amount were statistically significant (facility type comparison *p* <0.001).

When out-of-pocket payments were compared for refugees and host-community members by facility type, the average payments were significantly lower for refugees in PHCCs (US$8, 95 % CI: 6.4–9.8 vs. $US17, 95 % CI: 12.3–21.9) and private clinics (US$28, 95 % CI: 23.7–31.5 vs. US$40, 95 % CI: 35.0–44.1) (refugee vs. host community comparison *p* <0.001 for both PHCCs and private clinics) and statistically similar for hospitals (US$58, 95 % CI: 37.0–78.5 vs. US $93, 95 % CI: 58.2–126.7; refugee vs. host community comparison *p* = 0.105 among hospital careseekers).

## Discussion

### Prevalence

Prevalence of NCDs differed significantly between adult Syrian refugees and Lebanese host community members. Significantly higher prevalence rates of hypertension, cardiovascular disease, and diabetes were observed in the host community whereas Syrian refugees had higher rates of chronic respiratory disease and arthritis. Hypertension was the most prevalent of the included NCDs for Lebanese host community members (10.6 %) and the second most prevalent, after arthritis, for refugees (7.6 %). However, hypertension prevalence estimates in this survey are significantly lower than those reported elsewhere in the literature, where regional prevalence has been estimated at 29.5 %, prevalence in Syria at 24.9 % and prevalence in Lebanon at 28.8 % [20–23]. Hypertension prevalence rates among adult Syrian refugees in Jordan have been estimated at 9.7 % using this same methodology [23]. The relatively small proportion of the population over age 40 in this survey is one potential explanation for the low prevalence rate. Another possible explanation is that the methodology relied on respondent reporting, where both undiagnosed cases and those cases that poorly understood their diagnosis would not have been captured (both of which can be indicators of lack of access to health information and healthcare services).

Diabetes was the other condition where prevalence estimates in this survey were significantly lower than estimates reported by other sources. Diabetes prevalence has been estimated at 8.8 % in Syria and 14.9 % in Lebanon [24], which compares to survey estimates of 7.9 and 5.2 % for Syrians and the Lebanese host community, respectively. Undiagnosed cases, different case definitions and estimation methods, selective refugee migration and a non-representative Lebanese sample may be reasons for the observed differences, however it is likely that true disease burden for both hypertension and diabetes is underestimated in this survey. With respect to observed prevalence rates of other conditions, of cardiovascular disease has been estimated at 5.8 % in Syria and chronic respiratory conditions at 5.3 % in Lebanon and 6.1 % in Syria [25]; point estimates in the survey were slightly below these figures, however, differences are not substantial.

### Care-seeking and health service utilization

Overall, refugees accessed care for NCDs at a similar frequency as Lebanese host community members, suggesting they are able to receive needed care. Regression analysis of household characteristics for both refugees and Lebanese host community factors did not identify any predictors that suggested that certain types of households were less able to access medical care for NCDs. For all NCDs, refugees most often sought care in the PHCCs (60 %) whereas Lebanese host community members most often sought care in private clinics (63 %). Refugees were more likely to seek care in pharmacies (9.5 % versus 2.8 % host community) which could be detrimental to long term health if they are bypassing care from clinicians; however, given the relatively low out-of-pocket payments reported by refugees in the PHCCs, there may be other reasons such as location, availability of medicines, perceptions of quality of care, or other factors that contribute to the higher use of pharmacies.

Overall, refugees were less likely to seek care at hospitals than Lebanese host community members (9.2 % versus 2.8 %); cardiovascular disease was the only condition where use of hospital care among refugees (24.5 %) exceeded that of host community members (22.1 %). Among refugees, cardiovascular cases reported the lowest proportion utilizing primary health care centers for care (44.9 %) but the highest proportion utilizing hospitals (24.5 %). Payments among refugee hospital users were significantly higher among those with cardiovascular disease, with a mean payment of $US98 (median US$33) compared to a mean of US$58 (median US$20) for all conditions. It is possible that refugee hospital expenditures for cardiovascular disease reflect differences in presentation between refugees and host community members, where refugees are presenting with more complicated and advanced cases. This could be due to inability to afford medications and poor adherence, which can lead to adverse events, poor quality of care and/or delayed care-seeking where out-of-pocket payments are large in comparison to the households’ discretionary income.

However, overall, both the proportion of refugees with out-of-pocket payments and payment amounts were lower among refugees than host community members, suggesting that current humanitarian support is contributing to maintaining refugee access to NCD care. This finding is in contrast to recent survey of Syrian refugees in Lebanon which found that 56.1 % of households had a member with an NCD that was unable to access medicine or other services which suggest that situation may be more complicated, in particular with respect to medicines; cost was the primary barrier to care reported there and also in our survey [26]. One possible reason for the observed difference is that the UNHCR health access survey combines access to care and medicines and reports at a household level whereas data presented here focuses on individual care-seeking. Findings from this survey demonstrate that Syrian refugees in Lebanon are under considerable financial stress. On all economic measures, they are worse off than their Lebanese host community counterparts despite universal humanitarian assistance coverage. However, as funding for the Syria Crisis falls short of needs in the region, further reductions in assistance are anticipated which likely result in cost becoming an even greater barrier to care-seeking [27].

### Limitations

With respect to sampling, reliance on UNHCR registration data may have resulted in sampling bias if the geographic distribution of registered and unregistered households differed. Reallocation of clusters in areas controlled by militarily and political factions where permission to conduct the survey was not secured, specifically in the South, Southern suburbs of Beirut, and Northern areas of Bekaa, resulted in large area of the country being excluded. The survey coverage area included only 53 % of registered Syrian refugees and thus is not representative of the entire Syrian refugee population in Lebanon. In some of these areas, in particular South Beirut and North Bekaa, access to health services is perceived as particularly poor, thus survey findings may present a better picture of health access than if the entire country was included. The within cluster referral process presents the potential for bias, as respondents may not have always referred to the nearest household; referral procedures and small clusters size may have attenuated within-cluster similarities and the associated design effect. Replacement sampling, which was done for logistical purposes, could have contributed to bias if there were systematic differences between households with no one home compared with those interviewed. Additionally, the Lebanese host community sample was selected using a neighborhood approach and is reflective of those communities hosting the greatest number of refugees. As such, findings on the Lebanese host community population should not be generalized to the Lebanese population. Interviews were conducted by Lebanese, which could have resulted in a higher refusal rate or influenced refugee responses to certain questions such as income. Finally, the 2:1 ratio of refugee to host community households in some instances yielded inadequate sample size for statistical comparisons. Finally, it is possible that some of the differences found between refugees and host communities could be attributed to age differences; however, age-adjusted analysis was not possible due to the fact that age was categorically measured and specific age data were not collected for the one randomly selected individual with each NCD from a household answering the questions about care-seeking for that condition.

## Conclusions

NCDs are of growing importance in refugee populations, as an increasing proportion of refugees are from middle income countries with high NCDs burdens. The Middle East has a high NCD burden, and both Syrian refugees and the Lebanese host community are no exception. For refugee populations living in non-camp settings, such as those in Lebanon, this translates to immense burden on the health system. Given the protracted nature of the crisis, the high costs of providing NCD care and the large caseload of Syrian refugees with NCDs, implications for the Lebanon’s health system are immense. The burden of out of pocket expenses on persons with NCDs are also substantial, especially given the tenuous economic status of many refugees and the less affluent segments of the Lebanese population. While health service utilization rates were relatively similar for refugees and host community members, care-seeking location and out-of-pocket expenditures differed greatly between the two groups where refugees favored the public sector and Lebanese more often sought care in the private sector. Lebanon’s health system is largely privatized and the private sector is poorly regulated; the public sector is over-burdened and the perceived quality of care is lower. Greater investment in the public sector health system, including supply of medications for NCDs, could benefit both Lebanese and Syrian refugees alike. Efforts to improve quality of care for NCDs, such as the Ministry of Public Health’s recent update of treatment guidelines, are also a critical component of preventing adverse outcomes and lowering the cost of care for NCDs. Going forward, attention to the sustainability of these different efforts will be critical, as international humanitarian assistance funding which has helped to support the health sector, will eventually be curtailed and the Government of Lebanon will need to find a sustainable long term approach to addressing the increasing burden of NCDs.

## Abbreviations

ITS, informal tented settlement; NCDs, non-communicable disease; PHCC, primary health care center; UNHCR, United Nations High Commissioner for Refugees

## Footnotes

1 Reported age-specific prevalence is weighted by the number of household members in each age group. Adult prevalence is weighted by the number of adult household members reported.
